# Cat-to-Human Orthopoxvirus Transmission, Northeastern Italy

**DOI:** 10.3201/eid1503.080813

**Published:** 2009-03

**Authors:** Fabrizio Carletti, Licia Bordi, Concetta Castilletti, Antonino Di Caro, Laura Falasca, Cristiana Gioia, Giuseppe Ippolito, Stefania Zaniratti, Anna Beltrame, Pierluigi Viale, Maria Rosaria Capobianchi

**Affiliations:** National Institute for Infectious Diseases “L. Spallanzani,” Rome, Italy (F. Carletti, L. Bordi, C. Castilletti, A. Di Caro, L. Falasca, C. Gioia, G. Ippolito, S. Zaniratti, M.R. Capobianchi); University of Udine, Udine, Italy (A. Beltrame, P. Viale)

**Keywords:** Orthopoxvirus, zoonosis, human infection, letter

**To the Editor:** Kurth et al (*1*) recently described a cowpoxvirus chain of transmission from rat to human through an elephant in Germany. Zoonotic cowpoxvirus infections are well known in Europe (*2*,*3*). This virus can infect many animal species; serologic evidence of infection may approach 10% in cats in western Europe (*4*,*5*). Zoonotic orthopoxvirus (OPV) infection has been reported in several European countries, but it is rare south of the Alps, and no extensive description of cases is available for Italy. We describe 2 cases of zoonotic OPV in Friuli, northeastern Italy, in veterinary personnel scratched by cats.

In December 2005, a male veterinary student (patient A) who had been scratched by a cat with multiple cutaneous ulcerated lesions sought care at a local hospital; he had a lesion on his right hand, moderate fever, and malaise. Histopathologic findings from the cat indicated feline poxvirus infection. In July 2007, a female veterinarian (patient B) who lived in a different area of the same region and also had been scratched by a cat, sought care at the same hospital; she had a lesion close to the right sternoclavicular joint.

On the basis of patients’ history of exposure and clinical presentation of the animals’ disease, zoonotic transmission of OPV infection was suspected. Vesicle fluid and, subsequently, crusts from the patients’ lesions, were sent to the virology laboratory of the reference center for poxvirus infections at National Institute for Infectious Diseases in Rome, where OPV diagnosis was based on electron microscopy, virus isolation, and detection of viral nucleic acid. In addition, serial blood samples were sent to the same laboratory for analysis of specific antibodies and cellular immune response.

The viruses were almost identical, according to the partial sequence of the *crmB* gene (EF612710 and FJ445748) and on the complete sequence of the hemagglutinin gene (EF612709 and FJ445747). The hemagglutinin (Figure) and the partial *crmB* (not shown) sequences from each isolate formed a distinct cluster within the OPV genus; similar results were obtained from concatenated analysis of both genes (not shown). The identity/similarity scores of the complete nucleotide sequences of *crmB* and hemagglutinin genes from patient A, in relation to other OPVs, were, respectively, Ectromelia (AF012825), 0.598 and 0.841; cowpox (X94355), 0.933 and 0.927; vaccinia (AY678276), 0.844 and 0.934; camelpox (AY009086), 0.941 and 0.940; monkeypox (DQ011153), 0.909 and 0.914; and variola (DQ437588), 0.922 and 0.906. On the basis of these results, species assignment of the isolated OPVs was not possible. Preliminary sequence data on additional genes (ATI, A27L, and CBP) from patient A’s isolate supported the segregation of these OPVs in Italy from the other known OPV species. However, the available information is still not enough to infer whether the isolates from Italy belong to a novel or known OPV species. More extensive biologic and molecular characterization is in progress.

**Figure:**
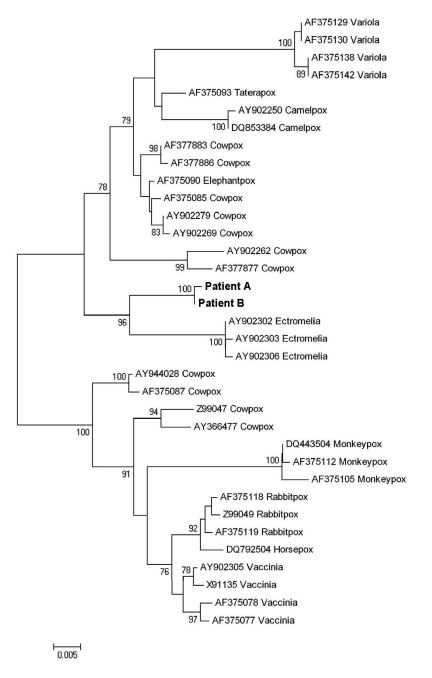
Phylogenetic tree of nucleotide sequences of the complete hemagglutinin open reading frame (930 bp) of orthopoxviruses (OPVs) isolated from the 2 patients described in this report and additional poxviruses available in GenBank (cowpox: AY902279, AY902269, AF375085, AF377883, AF377886, AY902262, AY944028, Z99047, AY366477, AF377877, AF375087; taterapox: AF375093; camelpox: AY902250, DQ853384; horsepox: DQ792504; elephantpox: AF375090; vaccinia: AY902305, X91135, AF375078, AF375077; rabbitpox: AF375119, AF375118, Z99049; variola: AF375129, AF375130, AF375138, AF375142; ectromelia: AY902302, AY902303, AY902306; monkeypox: DQ443504, AF375105, AF375112). Multiple alignment was generated with ClustalW 1.7 software in BioEdit (www.mbio.ncsu.edu/BioEdit/BioEdit.html), and the phylogenetic tree was constructed by using maximum-likelihood and neighbor-joining algorithms implemented in Mega 4.0 software (www.megasoftware.net). Bootstrap values >75 are shown at nodes. The sequences from the patients described in this report form a distinct cluster separate from other OPV sequences. Scale bar indicates genetic distance.

The 2 cases reported here, occurring >1 year apart, indicate that OPV is circulating in domestic, and possibly wild, local fauna; they underscore the need for physicians and veterinarians to become aware of the risk for OPV zoonoses. A surveillance program has been launched among local veterinary clinics to identify nonvaccinated veterinarians who have been exposed to OPV. A surveillance plan for cats at these clinics has also been started.
